# Blood Coagulation Factor IX: Structure, Function, and Regulation

**DOI:** 10.1002/iub.70024

**Published:** 2025-05-22

**Authors:** Lacramioara Ivanciu, Rodney M. Camire

**Affiliations:** ^1^ Division of Hematology and the Raymond G. Perelman Center for Cellular and Molecular Therapeutics The Children's Hospital of Philadelphia Philadelphia Pennsylvania USA; ^2^ Department of Pediatrics, Perelman School of Medicine University of Pennsylvania Philadelphia Pennsylvania USA

**Keywords:** factor IX. IXa, hemostasis, regulation, zymogen‐to‐protease transition

## Abstract

Blood coagulation factor IX plays a crucial role in the intrinsic pathway of coagulation by generating factor Xa, ultimately leading to thrombin formation. Over the past 50 years, extensive research has deepened our understanding of the biology, physiology, pathology, biochemistry, and molecular genetics of factor IX. This wealth of knowledge has revealed how the factor IX gene and protein evolved, how factor IX is regulated, how it functions within the coagulation cascade, and how structural changes affect its function. In this review, we will summarize current knowledge on the biology of factor IX, with a focus on its structure–function relationships, gene structure, and regulation.

## 1 Introduction

The blood coagulation response has evolved to rapidly form a localized clot and prevent blood loss following vascular injury. This finely regulated system consists of a network of serine proteases, their cofactors, and inhibitors. Most coagulation proteins circulate as inactive precursors that require limited proteolysis to become active enzymes. For many, full catalytic activity is only achieved when these enzymes assemble with their cofactors on an anionic membrane surface, effectively localizing the reaction to the site of injury [1, 2]. These proteins participate in a cascade of sequential proteolytic reactions that ultimately convert prothrombin into thrombin, the key effector of the coagulation cascade. In addition, plasma inhibitors are essential to downregulate these activated proteins once their function has been fulfilled, ensuring tight control of the system [2].

Coagulation factor IX (FIX) is part of the vitamin K–dependent family of coagulation factors—including prothrombin, factor VII (FVII), factor X (FX), protein C (PC), protein S, and protein Z—all of which share similar domain structures and significant sequence homology. In plasma, FIX circulates as an inactive zymogen and is activated to FIXa during coagulation. Unlike most serine proteases in this family, FIXa on its own has limited enzymatic activity. Its full catalytic potential is achieved upon assembly with its cofactor, factor VIIIa (FVIIIa), on phospholipid membranes, where it converts FX to FXa in the intrinsic pathway [1]. Naturally occurring mutations that reduce FIX activity or expression result in the bleeding disorder hemophilia B (HB), while gain‐of‐function mutations, such as FIX‐Padua, are associated with an increased risk of thrombosis [3, 4].

## 2 Historical Perspective

**FIGURE 1:**
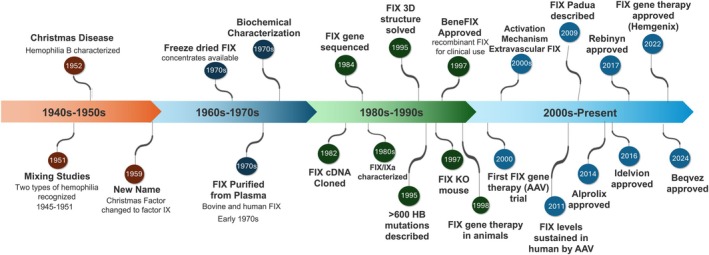
History of FIX characterization. Overview of key milestones in FIX biology, physiology, biochemistry, clinical assessment, and therapeutic development.

The first recognition of human hemophilia appears in 2nd‐century AD Jewish writings, though the clinical syndrome was not formally defined until the 19th century. The term *hemophilia* (meaning “love of blood”) was introduced in 1828 [5]. Hemophilia has often been referred to as the “royal disease,” as Queen Victoria of England (1837–1901) was a known carrier of HB [6]. Until the early 1940s, the role of FIX in blood clotting was not understood. It was only in the late 1940s (see Figure 1) that mixing studies demonstrated that blood from one hemophilia patient could correct the clotting deficiency of another, suggesting distinct types of hemophilia [7]. In 1952, using this approach, Biggs and colleagues identified a family with a condition clinically like hemophilia A but distinguishable through mixing studies. This disorder was named Christmas disease (HB), after the first patient described [8].

Throughout the 1950s and 1960s, research focused on elucidating the role of FIX in coagulation and its relationship to HB. Due to technological limitations, early studies relied on developing assays and analyzing FIX in plasma. In the 1970s, researchers were able to purify FIX from bovine and human plasma, leading to rapid advances in understanding its structure, function, and amino acid sequence [9]. By the 1980s, the human FIX cDNA was isolated and sequenced, the FIX gene was cloned, and key biochemical properties were identified [10, 11]. These breakthroughs enabled the recombinant production of FIX, driving research into its molecular structure, function, and the genetic causes of HB. Clinically, this knowledge led to the approval of recombinant FIX as the second recombinant clotting factor (after FVIII) in 1997 and culminated in the approval of the first clotting‐factor gene therapy product in 2022. Gene therapy for HB largely centers on strategies to achieve therapeutically relevant FIX activity levels with the lowest possible vector dose. Factor IX‐Padua, a variant that harbors a single amino acid substitution (R338L), has approximately 8‐fold increased specific activity [12]. The use of this gain‐of‐function mutant has led to major advances in gene therapy to treat HB, with two FDA‐approved AAV gene therapy products (Hemgenix, 2022 and Beqvez, 2024) now available to patients [13].

## 3 FIX Structure

**FIGURE 2:**
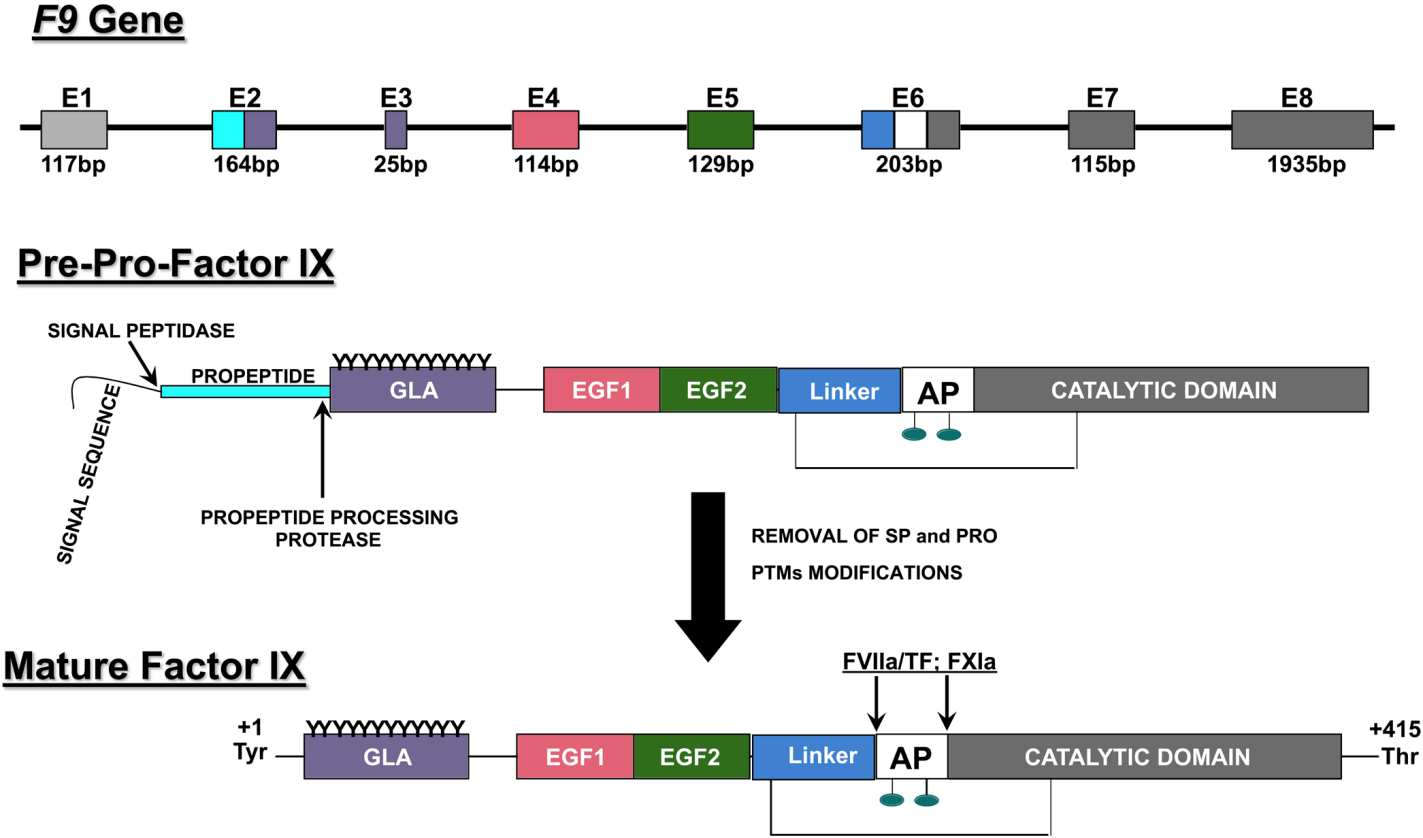
Structural organization of human *F9* gene and FIX protein. The F9 gene comprises 8 exons and 7 introns and 34 kb long. The number and length of each exon is provided and are color coded for each domain they encode. Pre‐pro‐FIX and mature FIX are shown with domains annotated along with specific cleavage sites by physiological activators TF/FVIIa and FXIa. Starting from the N‐terminus, the signal peptide (S), followed by the propeptide (P), the GLA domain, and epidermal growth factor domains (EGF), linker connects the activation peptide (AP). The C‐terminus contains the serine protease domain. The legacy numbering system: The +1 codon refers to the first amino acid of the mature protein and the last amino acid is 415.

The *F9* gene is 34 kb in length and is located on the X chromosome at band Xq27.1 [10, 14]. It consists of 8 exons and 7 introns. Exon 1 encodes the signal peptide; exon 2 encodes the propeptide and the γ‐carboxyglutamic acid (Gla) domain; exon 3 encodes the remainder of the Gla domain along with a short hydrophobic sequence. Exons 4 and 5 encode the two epidermal growth factor (EGF) domains; exon 6 encodes the linker region, activation peptide, and part of the catalytic domain; and exons 7 and 8 encode the remaining portions of the catalytic domain (Figure 2).

Factor IX is a vitamin K–dependent glycoprotein synthesized in the liver [15]. It is produced as a 461–amino acid preproprotein (prepro‐FIX) that includes several distinct regions (Figure 2). This structure consists of a 28‐residue signal peptide, which directs the nascent protein into the endoplasmic reticulum (ER), and an 18‐residue propeptide that facilitates interaction with vitamin K–dependent γ‐glutamyl carboxylase [16, 17]. Maturation of FIX requires the removal of both the signal peptide and propeptide, along with multiple post‐translational modifications. These include γ‐carboxylation of 12 glutamic acid residues, glycosylation, hydroxylation at residue 64, and phosphorylation [17, 18].

The mature form of FIX present in plasma is a single‐chain protein of 415 amino acids with a molecular mass of approximately 57,000 Da. It is composed of several domains: an N‐terminal Gla domain (amino acids 1–40), a short hydrophobic sequence (aa 41–46), two EGF domains (EGF‐1: aa 47–83; EGF‐2: aa 88–125), a linker region (aa 126–145), an activation peptide (aa 146–180), and a C‐terminal serine protease domain (aa 181–415) (Figure 2). Calcium (Ca^2+^) ions are essential for the proper folding of the Gla and EGF domains, with well‐characterized calcium‐binding sites [19, 20]. Additionally, 11 disulfide bonds stabilize the overall structure of the FIX molecule.

## 4 Domain Structure of FIX

**FIGURE 3:**
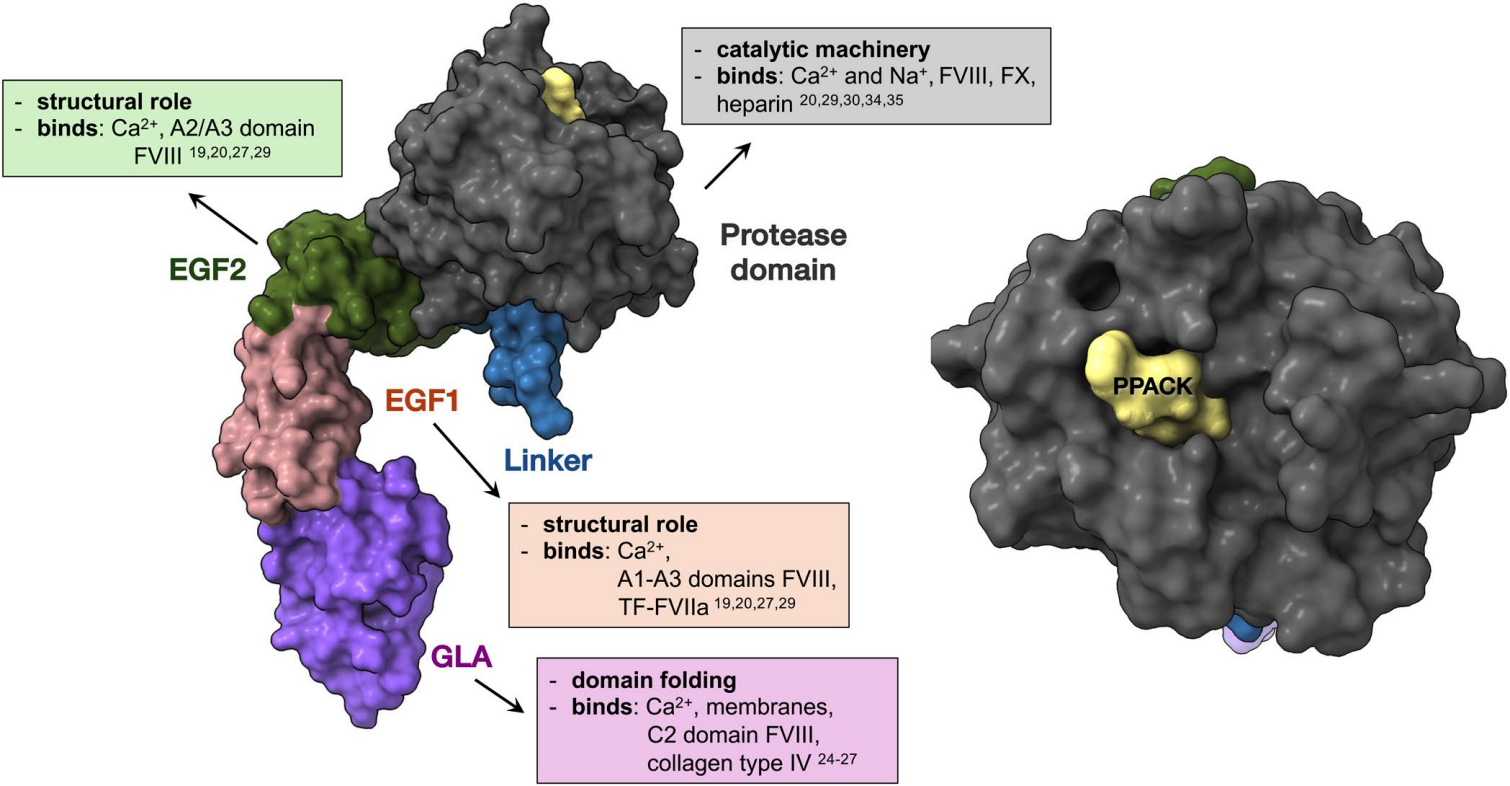
Factor IXa 3D structure. The surface rendered structure of porcine FIXa (1PFX) is shown with the active site inhibitor PPACK in the active site. FIXa domains and their functional properties are shown: The Gla domain is colored purple, EGF1‐pink, EGF2‐green, linker‐blue, and the protease domain is colored grey. The FIXa inhibitor PPACK is colored yellow. A close‐up view of the protease domain is shown on the right with PPACK indicated and the rest of the molecule is out of view in the back.

The three‐dimensional structure of full‐length porcine FIXai (Figure 3) was first determined in 1995 [21]. Currently, more than 25 structures of FIXa, primarily from human sources, are available in the RCSB Protein Data Bank (rcsb.org). Approximately half of these structures were deposited in 2010 or earlier.

### 4.1 The Gla Domain

The Gla domain of FIX (residues 1–40) contains 12 γ‐carboxylated glutamic acid (Gla) residues. The first 11 Gla residues are highly conserved among other vitamin K–dependent proteins and are critical for function, while the last two appear to have minimal functional significance [22]. γ‐Carboxylation of these glutamic acid residues occurs in the ER, catalyzed by a γ‐glutamyl carboxylase enzyme that recognizes and binds the propeptide of FIX [16, 23]. Vitamin K acts as a cofactor in this reaction, with vitamin K–epoxide reductase recycling vitamin K to its active form. The Gla domain binds multiple Ca^2+^ ions, which are essential for proper domain folding and for enabling interactions with phospholipid membranes [24]. Calcium binding is critical for FIX activity. In addition to membrane binding, the Gla domain also contributes to interactions with the C2 domain of FVIIIa and with collagen IV [25, 26, 27].

### 4.2 The EGF Domains

The EGF domains (EGF‐1, residues 47–83; EGF‐2, residues 88–125) of FIX are characterized by a distinct disulfide bond pattern which gives the domain considerable stability [28]. EGF1 binds Ca^2+^ (residues 47, 48, 50, 64 and 65) and also contains β‐hydroxyaspartic acid at position 64 and an O‐linked glycosylation site at positions 53 and 61 [19]. EGF domains play a structural role by enabling space between the protease domain and membranes which is crucial as FIXa needs to bind FX and FVIIIa, which are also bound to the membranes. EGF domains also mediate protein–protein interactions. Biochemical and molecular modeling studies support a role for the EGF domains in binding to FVIIIa [27, 29, 30]. In addition, EGF1 appears to mediate binding to the tissue factor–factor VIIa (TF–FVIIa) with Gly48 playing an essential role in FIX activation by the extrinsic pathway [29].

### 4.3 The Linker Region

The linker region (residues 126–145) connects the second EGF domain (EGF‐2) to the activation peptide (Figure 2). In the three‐dimensional structure of FIXa (Figure 3), this linker is positioned at the base of the protease domain. A key structural feature of the linker is a disulfide bond formed between Cys132 in the linker and Cys289 in the protease domain, which helps stabilize the protein structure. The final residue of the linker, Arg145, serves as one of the two cleavage sites for activation by factor XIa (FXIa) and TF–FVIIa complex; the other cleavage site is Arg180, located within the activation peptide.

### 4.4 The Activation Peptide

Although many coagulation zymogens—including prothrombin, FIX, FX, FXI, and PC—contain activation peptides, the sequences of these peptides are not conserved across proteins. The activation peptide of FIX (residues 145–180) is 35 amino acids long, has a molecular mass of approximately 11 kDa, and contains four glycosylation sites at residues 157, 159, 167, and 169 [31, 32]. Additionally, it is sulfated at residue 155 and phosphorylated at residue 158, both modifications enhancing protein recovery [17, 33]. The activation peptide is cleaved and removed during proteolysis at two specific sites: between residues 145–146 and 180–181, by the TF–FVIIa complex and FXIa [31]. As a result, the activation peptide is not present in the active form of the enzyme, FIXa.

### 4.5 The Protease Domain

The protease domain of FIXa (also known as the catalytic domain) spans residues 181–415 and shares high sequence and structural homology with other serine proteases (Figure 3). Its primary function is to provide binding surfaces for the cofactor FVIIIa and the substrate FX, and to catalyze cleavage of a specific peptide bond in FX. Structurally, the protease domain consists of two β‐barrels, each comprising roughly half of the domain. The substrate‐binding pocket and catalytic triad—composed of His57‐c, Asp102‐c, and Ser195‐c in the chymotrypsin numbering system, typically denoted with a “c” (corresponding to His221, Asp269, and Ser365 in legacy numbering system)—are located at the interface between these β‐barrels [34].

As noted earlier, Cys289 in the protease domain forms a disulfide bond with Cys132 in the linker region, covalently linking these two parts of the molecule. Beyond the active site, additional regions of the protease domain, known as exosites, play crucial roles in binding FX and FVIIIa. These exosites are primarily located in flexible loops and include features such as the 126‐c helix, 162‐c helix, and Asn178‐c. The protease domain also contains important ion‐binding sites: a single Ca^2+^ ion binds in the exosite I region, while a Na^+^ ion binds in the 186‐c/220‐c region. Exosite II is known to bind heparin and may also contribute additional binding surfaces for FVIIIa [29, 30, 35, 36].

## 5 FIX Activation

FIX circulates in the blood as an inactive, single‐chain zymogen, not known to be bound to other proteins. During coagulation, it is activated by physiological activators—TF/FVIIa complex and FXIa—which cleave FIX at two specific sites: Arg145 and Arg180. This cleavage produces the active, two‐chain, disulfide‐linked enzyme FIXa [31, 37].

Similar to other coagulation zymogens, cleavage at the conserved Arg180‐Val181 site (corresponding to Arg15c–Val16c in chymotrypsin numbering) unmasks a new N‐terminus on the heavy chain (Val16c‐Val‐Gly‐Gly19c) [38]. Initially, the cleaved molecule exists in a “zymogen‐like state”, but it rapidly transitions into the active protease form when the newly exposed N‐terminus inserts into the hydrophobic activation pocket. There, the Val16‐c residue forms an internal salt bridge with Asp194‐c [39, 40, 41, 42]. This interaction triggers a well‐defined conformational rearrangement that matures the active site, organizing the substrate‐binding pocket (S1 specificity site) and the adjacent oxyanion hole, both located near the catalytic residue Ser195‐c [41].

Although cleavage at Arg180 (Arg16‐c in chymotrypsin numbering) is sufficient to produce an intermediate form of FIXa capable of cleaving peptide substrates, full enzymatic activity requires a second cleavage at Arg145‐Ala146. This second cleavage, unique to FIX, releases the 35‐amino acid activation peptide [43]. Mutations associated with HB have been reported at both cleavage sites, underscoring the importance of both steps in generating fully functional FIXa [3, 44, 45].

## 6 Structure and Catalytic Properties of FIXa

FIXa is a two‐chain protein (∼45 kDa) composed of a covalently linked light chain (N‐terminal; residues 1–145) and heavy chain (C‐terminal; residues 181–415). The light chain (∼17 kDa) contains the Gla domain with 12 γ‐carboxylated glutamic acid (Gla) residues, two EGF domains, and a linker region. The heavy chain (∼28 kDa) contains the serine protease domain, which includes the catalytic triad (His57‐c, Asp102‐c, Ser195‐c), known as the active site cleft, and an oxyanion hole formed by Gly193‐c and Ser195‐c [34].

Compared with other coagulation proteases such as FXa and thrombin, FIXa exhibits relatively low catalytic activity and poorly cleaves small peptide substrates (e.g., Glu‐Gly‐Arg‐pNA) [36]. Its activity can be enhanced by the binding of molecules like Ca^2+^, Na^+^, and ethylene glycol, which induce conformational changes in the protease domain that improve catalysis [35, 46].

The reduced activity of FIXa is thought to result from partial obstruction of the active site entrance by residues Glu219‐c, Lys98‐c, and Tyr177‐c, collectively referred to as the “99‐loop.” [47] Mutations in this loop have been used experimentally to increase FIXa activity [35].

However, recent crystal structures of unbound (apo) FIXa and ligand‐bound FIXa complexes suggest that poor activity cannot be fully explained by loop 99 alone [20]. These structures revealed that the apo form of FIXa has a largely disordered active site, due to flexibility in the 215–225 polypeptide segment. Binding of an RNA aptamer (DTRI‐177) to an exosite region (comprising the 170‐and 220‐loops and residue Trp215) allosterically distorts the active site, locking FIXa into a closed, inhibited conformation. These studies demonstrate that FIXa exists in multiple conformational states, with significant movements of Trp215 controlling the opening and closure of the S1 pocket. This exosite‐mediated allosteric mechanism provides new insights into the regulation of FIXa activity and offers potential for therapeutic exploitation. In the presence of substrate, the S1 pocket opens, allowing improved catalytic function.

The physiological relevance of FIXa's low activity is incompletely understood. One explanation could be that FIXa has evolved to have limited activity as a negative regulatory mechanism against excessive coagulation. In support of this, several studies have linked high plasma levels of FIX(a) with increased risk of thrombosis [48].

## 7 Factor IXa and Intrinsic Xase

FIXa activates FX to FXa in the intrinsic pathway through proteolysis. However, FIXa alone is inefficient and requires additional components to effectively activate FX: Ca^2+^, negatively charged membranes (such as those on activated cells), and FVIIIa [1]. Membrane binding of FIXa and FVIIIa enhances the catalytic rate (*k*_cat_) of FX activation by at least 3000‐fold and reduces the reaction's binding affinity (K_m_) [1]. The assembly of the intrinsic Xase complex depends on the binding of these components to negatively charged membranes, with FIXa binding mediated by its Gla domain [24].

The interaction between FIXa and FVIIIa has been studied extensively. The protease domain of FIXa is thought to primarily mediate binding to FVIIIa, with additional contributions from the EGF2 domain [29]. Recent work by Childers et al. [27] confirmed these findings through computational protein–protein docking and small‐angle X‐ray scattering (SAXS), revealing that the FIXa–FVIIIa complex also involves interactions between the EGF1 domain of FIXa and the A1–A3 domains of FVIIIa.

The binding of FIXa to FVIIIa dramatically increases the rate of FX activation by approximately 20,000‐fold. In the context of the complete intrinsic Xase complex (FIXa, FVIIIa, membranes), the activity toward FX is enhanced by more than 1,000,000‐fold compared with FIXa alone [36]. These interactions between FIXa, membranes, FVIIIa, Ca^2+^, and FX together contribute to the extraordinary specificity and efficiency of FIXa within the intrinsic Xase complex, making it a key molecular engine in the coagulation system.

## 8 FIX and FIXa Clearance and Distribution

Clearance of FIX(a) from circulation occurs through multiple pathways, including binding to extravascular proteins, cell surface receptors, and endogenous inhibitors. In the bloodstream, FIX circulates at a concentration of approximately 5 μg/mL (90 nM) with a half‐life of 18–24 h [49]. In contrast, the ex vivo half‐life of FIXa in plasma is about 40 min—significantly longer than that of FXa, which has an ex vivo half‐life of less than 1 min [50, 51].

## 9 Extravascular FIX

In addition to circulating in the blood, FIX is also distributed in the extravascular space, where it binds specifically to collagen IV, a major component of basement membranes in all tissues [49]. This binding explains the rapid disappearance of FIX from circulation following infusion [52]. The amount of FIX in the extravascular compartment is higher than in plasma, with a rapid and reversible equilibrium existing between these two compartments [49].

FIX binds to collagen IV with high specificity and affinity (Kd ~5 nM), whereas other clotting factors—such as prothrombin, FX, or FVII—do not bind to collagen IV. The binding site, characterized in the late 1990s, is located in the Gla domain of FIX, specifically in the omega loop, with Lysine 5 (Lys5) playing a key role [26]. Mutations at this residue affect binding affinity: substitution with arginine increases binding, while substitution with alanine decreases it [49].

Preclinical studies suggest that extravascular FIX is biologically significant. For example, a knock‐in mouse model with reduced collagen IV binding (FIX K5A mouse) exhibits mild bleeding following tail transection and delayed vessel occlusion after ferric chloride‐induced injury of mesenteric arterioles—a model that exposes collagen IV [53]. Importantly, FIX‐K5A retains normal specific activity and phospholipid binding, indicating that the bleeding phenotype is specifically due to reduced collagen IV binding. Additional studies support the role of extravascular FIX in hemostasis. However, thrombus formation in the microcirculation appeared unaffected in K5A mice compared with wild‐type, suggesting that the importance of extravascular FIX may depend on the type of vessel and nature of the injury [53].

FIX is also able to bind collagen type I in vitro, suggesting a potential role of collagen type I in the distribution of FIX in the extravascular compartment [54]. However, experiments show that FIX, which accumulated in the knee joints of HB mice after multiple injections, colocalized mainly with collagen type IV and not type I.

Overall, these findings indicate that in mice, collagen IV–bound FIX plays a critical role in hemostasis, particularly in situations where collagen IV is exposed. To date, the role of extravascular FIX in preventing bleeding in humans has not been fully evaluated.

## 10 Inhibition by Endogenous Anticoagulants

FIXa activity is primarily regulated by the serpin antithrombin (AT), which targets the active site of FIXa [55, 56]. The conversion of FIX from its zymogen form to the active enzyme (following cleavage at Arg15c–Val16c) is essential for interaction with AT, as AT does not bind to the zymogen form. AT selectively inhibits FIXa via a classic suicide inhibition mechanism [56].

Mechanistically, AT uses its substrate‐like reactive center loop (RCL) to form a stable complex with the S1 pocket of FIXa's active site, with this interaction further stabilized by exosite contacts. Proteolytic cleavage at the P1–P1′ bond (per Schechter and Berger nomenclature [57]) within the RCL triggers a major conformational change in AT, driven by the insertion of the cleaved RCL into β‐sheet A of the serpin [58]. This structural rearrangement displaces the protease to the opposite side of AT, leading to the destruction of key catalytic features in FIXa, including the Val16c–Asp194c salt bridge and the oxyanion hole, effectively converting FIXa back into an inactive, zymogen‐like form [56, 58].

The rate of FIXa inhibition by AT is considerably slower than for other coagulation proteases—approximately 40‐fold slower than FXa and 125‐fold slower than thrombin [59, 60]. On the intact endothelial surface, AT activity can be accelerated by glycosaminoglycans such as heparan sulfates [61, 62]. Nevertheless, due to these relatively slow inhibition kinetics, the contribution of AT to FIXa regulation has historically been considered minimal in vivo.

However, our recent work [51] challenges this view. We identified that mutations at residues 16 or 17 of FIXa can shift the protease toward a more zymogen‐like state. Notably, our zymogen‐like variant, FIXa‐V16L‐c (V181L), displayed a ~ 20‐fold reduction in active site function [51]. Furthermore, FIXa‐V16L‐c was resistant to inhibition by plasma active‐site inhibitors such as AT. Interestingly, this reduced activity could be thermodynamically rescued through binding to FVIIIa on membrane surfaces. Using these zymogen‐like variants, we demonstrated that active‐site inhibitor regulation of FIXa plays a critical role in hemostasis in vivo. In HB mouse models, FIXa‐V16L‐c showed a significantly enhanced procoagulant response in several hemostatic challenges. For example, total blood loss in tail bleeding assays was significantly reduced over extended periods, and clot formation was more durable following ferric chloride‐induced or laser‐induced vascular injury. This represents the first in vivo experimental evidence that AT‐mediated inhibition of FIXa limits clot formation—findings that suggest translational potential for novel HB therapies.

While AT is the primary plasma inhibitor, other inhibitors may also contribute to FIXa regulation. These include protein Z‐dependent protease inhibitor (ZPI) [63] and protease nexin 2 (PN2) [64], both of which can form inactive complexes with FIXa. ZPI exerts its serpin inhibitory action through a mechanism like AT, whereas PN‐2 is presumed to target mainly the enzyme's active site. Additionally, FIXa activity may be modulated by protein S; mutations that disrupt the interaction between FIXa and protein S are associated with increased clot formation in venous injury models [65, 66]. Although the specific intermolecular contacts are not fully defined, the interaction may involve the EGF‐1 domain [65].

Lastly, FIXa catabolism in vitro is mediated by low‐density lipoprotein receptor‐related protein 1 (LRP1), a clearance receptor expressed on various cell types, including hepatocytes [67, 68]. Although LRP1 has been shown to bind FIXa both as a free enzyme and in complex with inhibitors, its physiological role in FIXa clearance in vivo has yet to be confirmed [67, 68, 69].

## 11 Concluding Remarks

Much effort has been directed towards elucidation of the biochemistry and molecular genetics of FIX over the past five decades. However, due to its critical role in the coagulation cascade and its clinical importance, FIX will continue to remain the center of intense research in the future. New research directions are expected to further elucidate the mechanisms of FIXa activity modulation and its interactions with cofactor FVIIIa or other proteins. Such knowledge will not only contribute to a fundamental understanding of the regulation of blood coagulation but may also expand our clinical understanding of bleeding and thrombosis and open new avenues for therapeutic development.

## Conflicts of Interest

Rodney M. Camire receives research support from Alnylam. Lacramioara Ivanciu—no conflicts of interest.
